# Supplementary material for: Nutrient availability induces community shifts in seagrass meadows grazed by turtles
Source: PeerJ. 2019 Sep 2;7:e7570. doi: 10.7717/peerj.7570 (PMC6727834; doi:10.7717/peerj.7570)
Supplement: Supplemental Information 1 — Late-successional species (Thalassia testudinum) and early-successional species (Syringodium filiforme + rhizophytic algae). Also included are average values ( +SE) of above and below-ground biomass and morphometric measurements of T. testudinum and S. filiforme. Levels: Control, Short-term, Medium-term, Long-term and Recovery. n = 5 in all levels (patches). *Levels with one or two outliers removed. [file peerj-07-7570-s001.docx]

|  | **Sample** | **Control** | **Short-term** | **Medium-term** | **Long-term** | **Recovery** |
| --- | --- | --- | --- | --- | --- | --- |
| **Density (ind m^-2^)** |  |  |  |  |  |  |
| *T. testudinum* | PVCring | 826 + 44 | 594 + 104 | 394 + 63 | 160 + 29 | 514 + 48 |
| *S. filiforme* | PVCring | 582 + 106* | 146 + 30 | 126 + 42 | 553 + 247* | 296 + 30* |
| Rhizoph. algae | PVCring | 114 + 53 * | 37 + 12 | 71 + 36 | 229 + 28* | 186 + 99 * |
| **Biomass (g DW m^-2^)** | |  |  |  |  |  |
| *T. testudinum* | core | 1161.5 + 73.6* | 774.7 + 88.2 | 658.3 + 66.2* | 220.1 + 73.4 | 253.6 + 66.9 |
| *S. filiforme* | core | 41.6 + 13.6* | 26.5 + 10.1 | 26. 9 + 9.9 | 44.3 + 22.8 | 12.1 + 7.4 |
| Rhizoph. algae | core | 65.4 + 35.6* | 8.1 + 5.5 | 13.2 + 10.3 | 6.9 + 4.5 | 22.2 + 22.2 |
| **Above-ground Biomass (g DW m^-2^)** | | |  |  |  |  |
| *T. testudinum* | core | 160.7 + 12.5* | 27.6 + 10.8 | 22.2 + 3.0* | 15.2 + 7.2 | 20.9 + 6.0 |
| *S. filiforme* | core | 2.7 + 1.2* | 1.2 + 0.5 | 0.5 + 0.4 | 3.3 + 1.6 | 5.6 + 4.8 |
| **Below-ground Biomass (g DW m^-2^)** | | | |  |  |  |
| *T. testudinum* | core | 1000.8 + 64.8* | 747.1 + 89.3 | 636.1 + 65.2* | 204.9 + 66.4 | 232.7 + 62.5 |
| *S. filiforme* | core | 39.0 + 13.3* | 25.4 + 9.6 | 26.4 + 9.6 | 41.1 + 21.3 | 6.6 + 4.9 |
| **Leaf dimensions (mm)** | | |  |  |  |  |
| *T. testudinum*  (width) | shoots  core | 10.88 + 0.31 | 8.63 + 0.22 | 6.70 + 0.16 | 6.71 + 0.13 | 6.65 + 0.14 |
| *S. filiforme*  (diam) | core | 1.34 + 0.09 | 0.93 + 0.11 | 0.89 + 0.05 | 1.10 + 0.04 | 1.02 + 0.09 |
| **Horizontal rhizome length (cm m^-2^)** | | |  |  |  |  |
| *T. testudinum* | core | 12392 + 1254 | 12608 + 1248 | 12806 + 3207 | 4298 + 675 | 5398 + 1256 |
| *S. filiforme* | core | 2449 + 1066 | 1308 + 490 | 1855 + 928 | 2604 + 1418 | 480 + 299 |

Appendix B. Average values (+ SE) and one-way ANOVA results used to test differences in the relative contribution of early-successional species to the total biomass of vegetation ((*S. filiforme* + rhizophytic algae) / (*S. filiforme* + rhizophytic algae + *T. testudinum*)). Values are average + SE. Letters indicate the groups resulting from pairwise comparisons.

|  |  | **Control** | **Short-term** | **Medium-term** | **Long-term** | **Recovery** | **ANOVA results** | | |
| --- | --- | --- | --- | --- | --- | --- | --- | --- | --- |
|  | **Sample** | **Mean + SE** | **Mean + SE** | **Mean + SE** | **Mean + SE** | **Mean + SE** | **df** | **F** | **p** |
| Relative contribution | PVCring | 0.35  + 0.08^a^ | 0.23  + 0.06^a^ | 0.21  + 0.08^a^ | 0.67  + 0.05^b^ | 0.36  + 0.10^a^ | 4 | 8.2385 | **<0.001** |
