# Supplementary material for: Nutrient availability induces community shifts in seagrass meadows grazed by turtles
Source: PeerJ. 2019 Sep 2;7:e7570. doi: 10.7717/peerj.7570 (PMC6727834; doi:10.7717/peerj.7570)
Supplement: Supplemental Information 2 [file peerj-07-7570-s002.docx]

|  |  | **Control** | **Short-term** | **Medium-term** | **Long-term** | **Recovery** | **ANOVA results** | | |
| --- | --- | --- | --- | --- | --- | --- | --- | --- | --- |
|  | **Sample** | **Mean + SE** | **Mean + SE** | **Mean + SE** | **Mean + SE** | **Mean + SE** | **df** | **F** | **p** |
| Relative contribution | PVCring | 0.35  + 0.08^a^ | 0.23  + 0.06^a^ | 0.21  + 0.08^a^ | 0.67  + 0.05^b^ | 0.36  + 0.10^a^ | 4 | 8.2385 | **<0.001** |
